# Heat stress affects mammary metabolism by influencing the plasma flow to the glands

**DOI:** 10.1186/s40104-024-01050-3

**Published:** 2024-07-05

**Authors:** Jia Zeng, Diming Wang, Huizeng Sun, Hongyun Liu, Feng-Qi Zhao, Jianxin Liu

**Affiliations:** 1 Institute of Dairy Science, College of Animal Sciences, Zhejiang University, Hangzhou, China; 2 Zhejiang Key Laboratory of Dairy Cow Genetic Improvement and Milk Quality Research, Zhejiang University, Hangzhou, China; 3 Department of Animal & Veterinary Sciences, University of Vermont, Burlington, VT USA

**Keywords:** Dairy cow, Heat stress, Mammary metabolism, Mammary plasma flow

## Abstract

**Background:**

Environmental heat stress (HS) can have detrimental effects on milk production by compromising the mammary function. Mammary plasma flow (MPF) plays a crucial role in nutrient supply and uptake in the mammary gland. In this experiment, we investigated the physiological and metabolic changes in high-yielding cows exposed to different degrees of HS: no HS with thermal-humidity index (THI) below 68 (No-HS), mild HS (Mild-HS, 68 ≤ THI ≤ 79), and moderate HS (Mod-HS, 79 < THI ≤ 88) in their natural environment. Our study focused on the changes in blood oxygen supply and mammary glucose uptake and utilization.

**Results:**

Compared with No-HS, the MPF of dairy cows was greater (*P* < 0.01) under Mild-HS, but was lower (*P* < 0.01) in cows under Mod-HS. Oxygen supply and consumption exhibited similar changes to the MPF under different HS, with no difference in ratio of oxygen consumption to supply (*P* = 0.46). The mammary arterio-vein differences in glucose concentration were lower (*P* < 0.05) under Mild- and Mod-HS than under no HS. Glucose supply and flow were significantly increased (*P* < 0.01) under Mild-HS but significantly decreased (*P* < 0.01) under Mod-HS compared to No-HS. Glucose uptake (*P* < 0.01) and clearance rates (*P* < 0.01) were significantly reduced under Mod-HS compared to those under No-HS and Mild-HS. Under Mild-HS, there was a significant decrease (*P* < 0.01) in the ratio of lactose yield to mammary glucose supply compared to that under No-HS and Mod-HS, with no difference (*P* = 0.53) in the ratio of lactose yield to uptaken glucose among different HS situations.

**Conclusions:**

Degrees of HS exert different influences on mammary metabolism, mainly by altering MPF in dairy cows. The output from this study may help us to develop strategies to mitigate the impact of different degrees of HS on milk production.

## Introduction

Global warming and heat stress (HS) have become significant challenges in livestock production. Environmental HS has been found to reduce milk production in mid-lactation cows by approximately 30% to 40% [1, 2]. One of the contributing factors to this reduction is a decrease in dry matter intake, which accounts for about half of the declined milk production [3]. However, the other underlying physiological and cellular mechanisms linking HS to reduced milk synthesis are not yet fully understood.

The synthesis of milk in lactating cows is dependent on the secretory capacity of the mammary gland (MG), including the number and activity of mammary secretory cells [4]. Studies have shown that exposure to high ambient temperatures leads to higher rates of programmed cell death in primary bovine mammary epithelial cells [5]. This increased cell death may contribute to the decrease in the total number of mammary epithelial cells and lower milk production observed in lactating cows under HS conditions [6]. Furthermore, HS affects mitochondrial protein oxidation and DNA loss [7], thereby impacting the energy metabolism of the body. Mammary plasma flow (MPF) plays a crucial role in nutrient supply and uptake in the MG [8] and is an important factor in supporting the synthesis of milk components. Despite the significance of MPF in providing nutrients for milk synthesis, limited research has been conducted on mammary nutrient uptake in animals under HS. Studies have shown that HS reduces MPF in mid-lactation dairy goats, leading to a decrease in net mammary glucose uptake [9]. Similarly, rabbits in early lactation experienced a 35% reduction in MPF when exposed to acute HS [10]. However, systematic studies investigating changes in MPF and mammary metabolism during HS are lacking in dairy cows.

We hypothesize that environmental HS affects mammary metabolism and milk synthesis in lactating cows by altering MPF and nutrient redistribution to the MG. In this experiment, we aimed to comprehensively examine the effects of HS on metabolism and milk synthesis in the MG of high-yielding dairy cows experiencing no HS to moderate HS. We focused on the changes in blood oxygen supply and mammary nutrient uptake and metabolism.

## Materials and methods

### Animals and experimental design

The experimental procedures involving the use of animals were approved by the Animal Care Committee at Zhejiang University (Hangzhou, China) and following the University’s guidelines for animal research. Eighteen high-yielding Chinese Holstein cows (milk yield = 41.4 ± 0.47 kg/d, days in milk = 207 ± 4.2 d, parity = 2–3; mean ± standard error) were selected and housed within the same barn in a dairy farm. They were subjected to three conditions of varying HS intensity: no HS with a temperature-humidity index (THI) below 68 (No-HS), mild HS (Mild-HS, 68 ≤ THI ≤ 79), or moderate HS (Mod-HS, 79 < THI ≤ 88) in their natural environment. The body temperature (39.7 vs. 38.8 vs. 38.4 °C) and respiratory rate (76.0 vs. 49.2 vs. 43.4 bpm) were significantly higher for the cows under Mod-HS vs. Mild-HS vs. No-HS [11]. Milk and blood samples were collected on three sampling days: May 15 (No-HS), June 18 (Mild-HS), and July 14 (Mod-HS) according to the change of THI. The details of feeding and management of the experimental cows have been described previously in our companion study [11]. All the cows were observed over a two-month period, and blood samples were collected from the coccygeal vein, caudal artery, and mammary vein of these cows 3 h after morning feeding on the day of sampling in all three conditions.

### Analyses of plasma metabolites and stress parameter

Blood physio-biochemical analysis was performed using a Clinical Analyzer (Model: 7020, Hitachi High-Tech Corporation, Tokyo, Japan). The concentrations of total protein (#B-2016), albumin (#B-2009), blood urea nitrogen (BUN, #C-3012), creatinine (#C-3007), glucose (#E-5003), non-esterified fatty acids (NEFA, #A-1011), triglyceride (#A-1008), cholesterol (#A-1007), high-density lipoprotein (HDL, #A-1005), low-density lipoprotein (LDL, #A-1006), alanine aminotransferase (ALT, #B-2010), aspartate aminotransferase (AST, #B-2011), alkaline phosphatase (ALP, #B-2012) in the coccygeal vein were measured using commercial kits from the Shanghai Juchuang Biotechnology Co., Ltd. (Shanghai, China). β-Hydroxybutyrate (BHB) was measured by using the commercial kit from the Zhongtuo Biological Co., Ltd. (Linyi, China). The concentration of cortisol (#H094-1-2), insulin (#H203-1-2), insulin-like growth factor-1 (IGF-1, #H041-1-2), glucagon (#H183), and glucagon-like peptide-1 (GLP-1, #H294-1) in the coccygeal vein were analyzed by commercial kits from the Nanjing Jiancheng Bioengineering Institute (Nanjing, China). The variables related to HS, hypoxia stress, and oxidative stress in mammary vein were also determined using commercial kits from the Nanjing Jiancheng Bioengineering Institute according to previously reported procedures [11]. Malondialdehyde (MDA, #A003-1-2) and total antioxidant capacity (TAOC, #A015-3-1) were determined by thiobarbituric acid reactants assay [12] and ferric reducing ability of plasma assay [13], respectively.

### Milk sampling and metabolite determination

In each period, milk yield was recorded for successive 3 d. Meanwhile, 50 mL of milk was taken daily at 3 time points (06:30, 13:00, and 19:00 h) and pooled for determination of milk composition (fat, protein, lactose, total milk solids, milk urea nitrogen, and somatic cell count). Glucose-related metabolites in milk, including pyruvic acid (#RXWB0070-96), hexokinase (#RXWB0125-96), 6-phosphofructokinase (PFK, #RXWB0140-96), citrate (#RXWB0156-96), lactic acid (#RXSH0511), and energy-related indexes (adenosine triphosphate, ATP, #RXWB0028-96) were determined using corresponding kits (Quanzhou Ruixin Science & Technology, Quanzhou, China).

### Calculations and statistical analysis

The MPF was estimated by Fick’s principle [14, 15] and calculated as below: MPF (L/d) = (milk phenylalanine + tyrosine) (g/d) × 0.965/[arterio-venous (AV) difference of (phenylalanine + tyrosine) (g/L)]. Concentrations of phenylalanine and tyrosine in the caudal artery, mammary vein, and milk were determined by an AA automatic analyzer (Hitachi High-Tech Technologies Corporation, Tokyo, Japan) as described previously [16].

Oxygen utilization in the MG was calculated as below:

1. Mammary oxygen supply (L/d) = MPF (L/d) × Caudal artery oxygen concentration (mL/dL)/100;
2. Mammary oxygen consumption (L/d) = MPF (L/d) × [Caudal artery oxygen concentration (mg/dL) − Mammary vein oxygen concentration (mg/dL)]/100;
3. Mammary oxygen utilization (%) = [Mammary oxygen consumption (L/d)/Mammary oxygen supply (L/d)] × 100%.

Glucose utilization by the MG was calculated with reference to previous study [17] and briefly described as below:

1. Mammary uptake of glucose (mol/d) = AV difference of glucose (mmol/L) × MPF (L/d);
2. Mammary clearance rate of glucose (L/h) = MPF (L/h) × AV difference of glucose (mmol/L)/Mammary venous concentration of glucose (mmol/L);
3. Mammary extraction rate of glucose (%) = AV difference of glucose (mmol/L)/Mammary arterial supply of glucose (mmol/L) × 100%;
4. Efficiency of mammary utilization of supplied glucose (%) = Lactose yield (g/d)/[Caudal arterial supply of glucose (g/L) × MPF (L/d)] × 100%;
5. Efficiency of mammary utilization of uptaken glucose (%) = Lactose yield (g/d)/[AV difference of glucose (g/L) × MPF (L/d)] × 100%.

To assess insulin resistance, the surrogate index was calculated according to Balogh et al. [18] by the equation: RQUICKI_BHB_ = 1/[log glucose (mg/dL) + log insulin (μU/mL) + log NEFA (mmol/L) + log BHB (mmol/L)], where RQUICKI_BHB_ represents the revised quantitative insulin sensitivity check index, BHB represents β-hydroxybutyrate, and NEFA represents non-esterified fatty acid. A lower value suggests greater insulin resistance.

Statistical analyses were performed using SAS 9.4 (SAS Inc., Cary, N.C., USA), and differences between treatments were analyzed using orthogonal polynomial comparisons with linear and quadratic effects, with treatment as fixed variables and individual cows as random variables. The statistical models were similar to the previous article [11]. The *P*-values for linear effect (Liner) and quadratic effect (Quadratic) were used, respectively. Histograms was made using GraphPad prism8 (San Diego, California, USA, www.graphpad.com). The results were presented as the mean and standard error of the mean (SEM).

## Results

### Mammary plasma flow and mammary oxygen metabolism

**Fig. 1:**
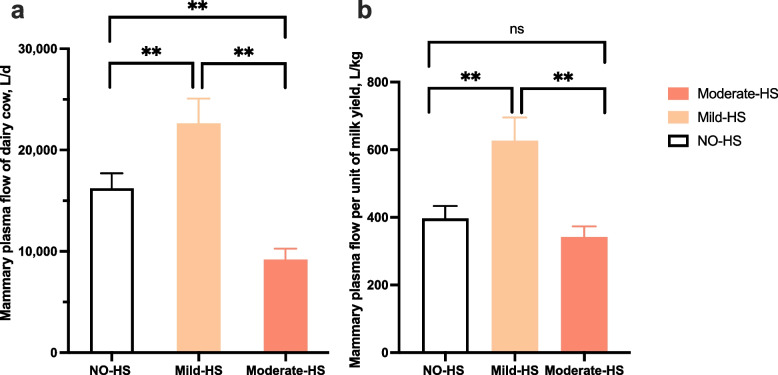
Mammary plasma flow per day (**a**) and per unit of milk yield (**b**) in dairy cows under different heat stress (HS). ** *P* < 0.01, ns* P* > 0.05

Figure 1 shows the MPF of dairy cows under different HS. Under Mod-HS, the MPF of dairy cows was lower (*P* < 0.01) than those under No-HS and Mild-HS. Under Mild-HS, overall MPF and the MPF per unit of milk yield were greater (*P* < 0.01) than those under No-HS and Mod-HS.

Oxygen metabolism parameters of dairy cows under different HS are presented in Table 1. Oxygen supply to the MG, overall mammary oxygen consumption, and oxygen consumption and supply per unit of milk produced in dairy cows under Mild-HS were significantly higher (*P* < 0.01) than those in cows under No-HS and Mod-HS conditions. Oxygen supply to the MG in dairy cows under Mod-HS was significantly lower (*P* < 0.01) than those under No-HS and Mild-HS. However, the ratio of oxygen consumption to oxygen supply in the MG of dairy cows did not show significant difference (*P* = 0.46) under Mild-HS and Mod-HS compared to the condition under No-HS.

**Table 1:** Mammary oxygen supply and consumption in dairy cows under different heat stress

Item	Heat stress			SEM	*P*-value		
	No	Mild	Moderate		Treat	Linear	Quadratic
O_2_ supply							
L/d	1,881^b^	2,504^a^	1,016^c^	190	< 0.01	< 0.01	< 0.01
L/kg milk	46.0^b^	69.2^a^	37.6^b^	5.14	< 0.01	0.26	< 0.01
O_2_ consumption							
L/d	329^b^	510^a^	213^b^	54.3	< 0.01	0.14	< 0.01
L/kg milk	8.03^b^	14.0^a^	7.92^b^	1.48	0.01	0.96	< 0.01
O_2_ utilization^1^	17.5	20.4	21.0	2.10	0.46	0.26	0.58

^1^ Mammary oxygen utilization (%) = [Mammary oxygen consumption (L/d)/Mammary oxygen supply (L/d)] × 100%

^a–c^ Means within the same row with different superscripts are different (*P* < 0.05)

### Plasma metabolites, biochemical parameters and hormones in coccygeal vein

Table 2 presents the plasma metabolites and biochemical parameters in the blood of coccygeal vein of dairy cows under different HS. Concentrations of total protein, creatinine, and globulin were greater (*P* < 0.01) under Mild-HS and Mod-HS than those under No-HS. Conversely, the concentration of BUN, AST, cholesterol, HDL, and LDL were lower (*P* < 0.01) under Mild-HS and Mod-HS than those under No-HS. The concentrations of ALT, ALP and glucose were lower (*P* < 0.01), whereas BHB concentration was higher under Mod-HS than those under No-HS and Mild-HS.

**Table 2:** Plasma metabolite and biochemical parameters in the plasma of coccygeal vein of dairy cows under different heat stress

Item^1^	Heat stress			SEM	*P*-value		
	No	Mild	Moderate		Treat	Linear	Quadratic
Protein metabolism							
Total protein, g/L	77.6^b^	79.9^a^	80.0^a^	1.22	0.03	0.02	0.22
BUN, mmol/L	6.60^a^	5.68^b^	6.00^b^	0.20	< 0.01	< 0.01	< 0.01
Creatinine, μmol/L	64.5^c^	66.8^b^	75.4^a^	1.73	< 0.01	< 0.01	< 0.01
Glucolipid metabolism							
Glucose, mmol/L	3.42^a^	3.42^a^	3.08^b^	0.06	< 0.01	< 0.01	< 0.01
NEFA, μmol/L	75.3	81.3	80.3	7.12	0.82	0.63	0.68
BHB, μmol/L	491^b^	530^b^	577^a^	26.3	< 0.01	< 0.01	0.83
Triglyceride, mmol/L	0.12	0.13	0.13	0.01	0.27	0.23	0.27
Cholesterol, mmol/L	7.25^a^	6.54^b^	5.01^c^	0.31	< 0.01	< 0.01	< 0.01
HDL, mmol/L	2.22^a^	2.13^b^	1.86^c^	0.06	< 0.01	< 0.01	0.01
LDL, mmol/L	1.34^a^	1.19^b^	0.94^c^	0.07	< 0.01	< 0.01	0.02
Liver function							
ALT, U/L	33.6^a^	32.7^a^	26.7^b^	0.89	< 0.01	< 0.01	< 0.01
AST, U/L	76.1^a^	70.8^b^	71.6^b^	3.20	0.01	0.02	0.06
ALP, U/L	32.2^a^	32.3^a^	23.2^b^	1.61	< 0.01	< 0.01	< 0.01
Albumin (A), g/L	35.2	35.2	35.2	0.30	1.00	0.95	0.99
Globulin (G), g/L	42.5^b^	44.7^a^	44.9^a^	1.28	0.04	0.02	0.24
A:G	0.84	0.80	0.80	0.03	0.12	0.07	0.33

^1^*BUN* Blood urea nitrogen, *NEFA* Non-esterified fatty acids, *BHB* β-Hydroxybutyrate, *HDL* High-density lipoprotein, *LDL* Low-density lipoprotein, *ALT* Alanine aminotransferase, *AST* Aspartate aminotransferase, *ALP* Alkaline phosphatase

^a–c^ Means within the same row with different superscripts are different (*P* < 0.05)

Hormones in coccygeal vein of dairy cows under different HS are shown in Table 3. Insulin concentration was significantly higher (*P* < 0.01) under Mild-HS compared to that under No-HS and Mod-HS. Conversely, glucagon concentration was lower (*P* < 0.01) under Mod-HS than those under No-HS and Mod-HS. Furthermore, there was a trend (*P* = 0.09) of higher ratio of insulin to glucagon during Mod-HS compared to other two conditions. Compared to Mild-HS, Mod-HS led to a significantly higher IGF-1 concentration (*P* < 0.01).

**Table 3:** Hormones in coccygeal vein of dairy cows under different heat stress

Item^1^	Heat stress			SEM	*P*-value		
	No	Mild	Moderate		Treat	Linear	Quadratic
Cortisol, ng/mL	47.9	46.47	44.2	1.81	0.13	0.05	0.78
Insulin, mIU/L	18.9^b^	22.0^a^	18.1^b^	0.90	< 0.01	0.39	< 0.01
Glucagon, ng/L	192^a^	200^a^	149^b^	8.95	< 0.01	< 0.01	0.01
Insulin:Glucagon	0.10	0.11	0.13	0.01	0.09	0.03	0.76
IGF-1, ng/mL	109^ab^	99^b^	114^a^	5.05	0.03	0.40	0.01
GLP-1, pmol/L	18.7	18.6	20.5	0.80	0.08	0.05	0.24
RQUICKI_BHB_	0.62	0.60	0.62	0.02	0.50	0.68	0.27

^1^*IGF-1* Insulin-like growth factor-1, *GLP-1* Glucagon-like peptide-1, *RQUICKI* Revised quantitative insulin sensitivity check index, RQUICKI_BHB_ = 1/[log glucose (mg/dL) + log insulin (μU/mL) + log NEFA (mmol/L) + log BHB (mmol/L)], *NEFA* Non-esterified fatty acids, *BHB* β-Hydroxybutyrate

^a,b^ Means within the same row with different superscripts are different (*P* < 0.05)

### Mammary glucose utilization and metabolism

Glucose utilization and metabolism in the MG of dairy cows under different HS are presented in Table 4. A significant increase (*P* < 0.01) in glucose concentration was found in mammary vein of cows under Mild-HS compared to that under No-HS. Conversely, a significant decrease (*P* < 0.01) in glucose concentration was found in the caudal artery of cows under Mod-HS compared to that under No-HS and Mild-HS. The mammary AV differences in glucose concentration (*P* < 0.05) and glucose extraction rates (*P* < 0.05) were lower under both Mild-HS and Mod-HS than those under No-HS. The glucose supply and flow were significantly increased (*P* < 0.01) under Mild-HS but significantly decreased (*P* < 0.01) under Mod-HS compared to those under No-HS. Under Mod-HS, both glucose uptake and clearance rates were significantly reduced (*P* < 0.01) compared to those under No-HS and Mild-HS. Under Mild-HS, there was a significant decrease (*P* < 0.01) in the ratio of lactose yield to mammary glucose supply compared to that under No-HS and Mod-HS, with no difference (*P* > 0.05) between No-HS and Mod-HS.

**Table 4:** Glucose utilization and metabolism in the mammary gland of dairy cows under different heat stress

Item	Heat stress			SEM	*P*-value		
	No	Mild	Moderate		Treat	Linear	Quadratic
Glucose concentration							
Artery (A), mmol/L	3.63^a^	3.60^a^	3.23^b^	0.09	< 0.01	< 0.01	0.04
Vein (V), mmol/L	2.64^b^	2.83^a^	2.52^b^	0.09	< 0.01	0.14	< 0.01
AV difference, mmol/L	1.00^a^	0.78^b^	0.71^b^	0.07	< 0.01	< 0.01	0.27
Glucose supply and utilization							
Supply (A), mol/d	58.4^b^	82.6^a^	29.4^c^	6.5	< 0.01	< 0.01	< 0.01
Flow (V), mol/d	42.2^b^	64.7^a^	22.9^c^	4.99	0.01	< 0.01	< 0.01
Uptake, mol/d	16.2^a^	17.9^a^	6.45^b^	1.95	< 0.01	< 0.01	0.01
Clearance rate, L/h	269^a^	262^a^	114^b^	34.77	< 0.01	< 0.01	0.11
Extraction rate, %	27.6^a^	21.3^b^	21.9^b^	1.97	0.02	0.02	0.09
Lactose/Supply^1^, %	21.2^a^	14.7^b^	25.1^a^	2.17	< 0.01	0.22	< 0.01
Lactose/Uptake^2^, %	68.3	61.1	72.9	6.24	0.53	0.66	0.27

^1^ Ratio of lactose yield to mammary glucose supply

^2^ Ratio of lactose yield to mammary glucose uptake

^a–c^ Means within the same row with different superscripts are different (*P* < 0.05)

Table 5 shows substances related to glucose metabolism in the milk of dairy cows under different HS. The ATP and citrate concentrations in milk significantly increased (*P* < 0.01) under Mild-HS and Mod-HS compared to that under No-HS. Concentration of lactic acid significantly decreased (*P* < 0.01) in milk under Mod-HS compared to that under No-HS and Mild-HS.

**Table 5:** Substances related to glucose metabolism in milk of dairy cows under different heat stress

Item^1^	Heat stress			SEM	*P*-value		
	No	Mild	Moderate		Treat	Linear	Quadratic
ATP, mmol/L	13.2^b^	19.1^a^	20.9^a^	1.01	< 0.01	< 0.01	0.11
Pyruvic acid, mmol/L	0.69	0.63	0.60	0.05	0.40	0.19	0.77
Hexokinase, mmol/L	0.20	0.21	0.17	0.02	0.26	0.21	0.28
PFK, μmol/L	87.6	93.9	100	8.77	0.60	0.32	1.00
Lactic acid, mmol/L	0.98^a^	0.94^a^	0.74^b^	0.05	0.01	< 0.01	0.19
Citrate, mmol/L	3.03^c^	3.91^b^	5.68^a^	0.17	< 0.01	< 0.01	0.04

^1^*PFK* Phosphofructokinase

^a–c^ Means within the same row with different superscripts are different (*P* < 0.05)

### Heat, oxidative, and hypoxia stress in MG

As shown in Table 6, the cows under Mild-HS and Mod-HS had greater values (*P* < 0.05) of mammary vein concentrations of nitric oxide (NO), heme oxygenase-1 (HO-1), TAOC, heat shock factor, vascular endothelial growth factor (VEGF), and heat shock protein 90 than those under No-HS.

**Table 6:** Variables in heat, oxidative, and hypoxia stresses in mammary vein of dairy cows under different heat stress

Item^1^	Heat stress			SEM	*P*-value		
	No	Mild	Moderate		Treat	Linear	Quadratic
NO, μmol/L	4.56^c^	6.63^b^	12.7^a^	0.44	< 0.01	< 0.01	< 0.01
iNOS, U/mL	12.7^a^	12.4^a^	11.3^b^	0.38	0.01	0.01	0.32
eNOS, ng/mL	4.82^a^	4.71^a^	4.27^b^	0.19	0.03	0.01	0.37
HO-1, ng/mL	17.7^b^	19.9^a^	19.9^a^	0.95	0.01	0.01	0.09
VEGF, ng/L	274^b^	301^a^	309^a^	10.9	0.01	< 0.01	0.34
HIF-1α, ng/L	225	241	227	9.86	0.11	0.84	0.03
MDA, nmol/mL	1.02^b^	1.33^b^	2.65^a^	0.13	< 0.01	< 0.01	< 0.01
SOD, U/mL	142^a^	128^b^	140^a^	4.95	< 0.01	0.48	< 0.01
GSH-Px, U/mL	105	105	94.1	4.38	0.14	0.10	0.27
TAOC, mmol/L	0.14^b^	0.18^a^	0.18^a^	0.01	< 0.01	< 0.01	0.09
HSF, ng/L	222^c^	267^b^	310^a^	9.88	< 0.01	< 0.01	0.87
HSP90, ng/mL	20.9^b^	23.1^a^	22.3^a^	0.78	0.01	0.05	0.02

^1^ *NO* Nitric oxide, *iNOS* Inducible nitric oxide synthase, *eNOS* Endothelial nitric oxide synthase, *HO-1* Heme oxygenase 1, *VEGF* Vascular endothelial growth factor, *MDA* Malondialdehyde, *SOD* Superoxide dismutase, *GSH-Px* Glutathione peroxidase, *TAOC* Total antioxidant capacity, *HSF* Heat shock transcription factor, *HIF-1α* Hypoxia inducible factor 1α, *HSP90* Heat shock protein 90

^a–c^ Means within the same row with different superscripts are different (*P* < 0.05)

The concentration of superoxide dismutase was lower (*P* < 0.01) under Mild-HS than under No-HS and Mod-HS, with no difference between No-HS and Mod-HS (*P* > 0.05). Additionally, the levels of hypoxia-inducible factor 1-alpha (HIF-1α) tended to be higher (*P* = 0.10) under Mild-HS compared to No-HS and Mod-HS.

Under Mod-HS, mammary vein concentrations of inducible nitric oxide synthase (iNOS) and endothelial nitric oxide synthase (eNOS) were lower (*P* < 0.05), while MDA level was higher (*P* < 0.01) under Mod-HS than under No-HS and Mild-HS.

## Discussion

Previous studies have highlighted the dependence of mammary nutrient utilization on MPF [19, 20]. Thus, it is essential to investigate the relationship between HS and blood flow in comprehending the impact of HS on mammary absorption. The present study specifically focused on MPF, mammary uptake of nutrients, milk synthesis, and MG homeostatic parameters in dairy cows under three levels of HS. We aimed to gain insights into the mechanism underlying the HS-induced milk production decrease.

Our study found that Mild-HS induced higher overall MPF and MPF per unit of milk yield, whereas Mod-HS resulted in a significantly lower MPF with the MPF per unit of milk yield unchanged due to lower milk yield (Fig. 1). Literature has also reported a tendency of HS to diminish MPF in mid-lactation goats [9]. Similarly, Lublin and Wolfenson [10] observed a 35% decrease in blood flow among non-pregnant rabbits during early lactation as a consequence of HS. In addition, other study in sows found that the right pudic artery blood flow increased under HS [21]. Furthermore, cows during HS experienced an increased cutaneous blood flow, facilitating heat dissipation from the core to the skin surface [22, 23]. The increased MPF during Mild-HS in this study may be required to meet the increased demands for lactation. Milk yield indeed decreased during Milk-HS in comparison with the NO-HS. However, the decrease in milk yield during Mild-HS may be attributed to both heat stress and lactation stage effects because the difference in lactation time from the NO-HS to Mild-HS was more than a month [11]. In a recent study, Hu et al. [24] found that milk yield of mid-lactating cows with high and low lactation persistency decreased on average by 1.84 and 3.50 kg per month, respectively. The decline (4.6 kg) from NO-HS to Mild-HS in our study [11] was not very large, suggesting that the cows during Mild-HS still strived to maintain the lactation through the enhanced MPF to increase nutrient supply. On the other hand, the increased respiratory rate usually resulted in an increase of heart rate, though we did not measure the heart rate. Under Mod-HS, blood redistribution may occur, with more blood flowing to the skin surface to promote heat dissipation, which may lead to a reduction in MPF along with a strong reduction in milk yield [11]. The regulation of MPF involves a complex interplay of positive and negative feedback mechanisms [25]. The NO has been known to play a role in regulating MPF [26], and elevated blood NO levels are associated with improved MPF in dairy cows [27, 28]. In the present study, we observed that the higher MPF in the MG of dairy cows under Mild-HS conditions was accompanied by elevating NO concentration in the mammary vein (Table 6). However, the MPF was significantly lower during Mod-HS compared to both Mild-HS and No-HS, although the mammary venous blood still exhibited much higher NO concentration, indicating that the blood NO levels are uncoupled with the MPF in the Mod-HS condition at least. Further studies are needed to investigate the relationship between MPF and NO in cows during HS. Similarly, VEGF activates angiogenesis [29] and may play an important role in regulating blood flow. The VEGF level was positively associated with MPF during Mild-HS, but not during Mod-HS in the present study.

Previous research has demonstrated that altering MPF could influence the uptake of the precursors of milk synthesis [30, 31], and HS may affect the oxygen availability in MG [11, 32]. Oxygen consumption has been considered as a marker of susceptibility to malignant hyperthermia and heat stroke [33]. Thus, in the current study, we assessed mammary oxygen consumption and utilization under varying degrees of HS. In comparison with No-HS, a substantial increase was found in both oxygen supply and oxygen consumption in the MG of dairy cows during Mild-HS, with a significant decrease in oxygen supply during Mod-HS. Combined with the changes in MPF, it is indicated that the changes in blood flow lead to significant changes in oxygen supply and oxygen consumption [34]. During lactation, possible low oxygen tension caused by increased metabolic rate and oxygen consumption may play a major role in stimulating glucose uptake and expression of glucose transporter 1 (GLUT1) in mammary epithelial cells [35, 36]. It is worth noting that oxygen plays a pivotal role in maintaining cellular metabolism within normal parameters. The HS induces a general decline in mammary tissue metabolic activity, particularly affecting carbohydrate and lipid metabolism [36]. The HS may decrease mammary metabolic rate and cost energy for homeostatic regulation. The cows during Mild-HS increased oxygen consumption may be not only for enhancing milk synthesis but also for additional physiological demands in response to heat stress per se such as temperature regulation, etc. Thus, the increase in MPF may not compensate for the demand for milk synthesis in the mammary gland, resulting in a slight decrease in lactation performance [11]. However, the supply of milk synthesis precursors may largely decrease due to the decreased MPF under Mod-HS, leading to a highly decreased lactation performance [11].

During lactation, the uptake of glucose by the MG is essential for lactose synthesis [37], as the synthesis of lactose and other milk components rely on precursors primarily obtained through the circulatory system [30]. Our previous study found the lactose yield during Mild- and Mod-HS are significantly lower than in No-HS, with significantly lower value for Mild-HS than for No-HS [11]. Lactose acts as the primary osmotic factor driving milk production [38]. Existing literature suggests that heat-stressed cows experience downregulation of glucose transporter proteins and reduce utilization of glucose for lactose synthesis [39, 40], thereby also affecting mammary lactose synthesis. Heat-stressed cows also preferentially utilize glucose as an energy source and reduce mobilization of triglyceride from adipose tissue, which can maximize glucose utilization in the MG and hepatic glucose output [2]. In the present study, the glucose supply to the MG was increased, but the glucose uptake was not changed due to decreased extraction rate during Mild-HS (Table 4). However, the glucose supply, uptake, and extraction rate were all significantly lower in Mod-HS. Nevertheless, the ratio of lactose to glucose uptake was not changed in both Mild-HS and Mod-HS conditions, supporting the previous consistent finding that the glucose uptake in the MG is linearly or positively associated with lactose or milk production [35]. The expression of the GLUT significantly changes under HS [40]. This change in expression may also be associated with the decreased glucose uptake observed during HS.

Mammary glucose metabolism is influenced by body metabolism and hormonal regulation. Heat-stressed animals often experience a lack of fat and triglyceride mobilization [41, 42]. Consistently, in our study, we observed significantly lower concentrations of BUN, HDL, LDL, and cholesterol in the coccygeal vein of dairy cows during both Mild- and Mod-HS and lower concentrations of glucose during Mod-HS. Previous studies have shown that cows under HS preferentially use glucose as energy [2]. Indeed, the arterial glucose concentration was lower, while the coccygeal vein BHB concentration was higher in Mod-HS, indicating that when glucose supply is insufficient, heat-stressed cows also carry out fat mobilization, resulting in increased BHB concentration. These changes may be associated with reduced feed intake and hormonal regulation [5]. Insulin and glucagon play a crucial role in this process. Insulin is a major anabolic hormone that controls key energy functions, including glucose and lipid metabolism [2, 43]. The role of glucagon in ruminants is involved in maintaining the basal rate of gluconeogenesis, and previous study has shown that glucagon is decreased during HS [44]. Interestingly, we observed a higher insulin concentration during Mild-HS, but a lower concentration of glucagon during Mod-HS, suggesting different roles or different regulations of these two hormones during two different HS conditions. During Mild- and Mod-HS, ATP and citric acid concentrations in milk were significantly higher compared to conditions without HS. The increased citrate levels in milk also indicate MG dysfunction [45]. It is worth noting that our blood observations were at 3 h after feeding which may have an impact on the values of these observations, but the blood samples were taken at the similar times among groups. Anyhow, the sampling time may have an impact on body physiology, such as insulin resistance. Overall, our findings highlight the intricate relationship among hormonal regulation, nutrient supply, body, and mammary metabolism in dairy cows during the HS.

Heat stress affects mammary homeostasis, which changes the normal physiological functions of the MG. Excessive NO levels observed in HS conditions may trigger lipid peroxidation, as evidenced by a significant increase in MDA concentration in mammary veins (Table 6), an indicator of the occurrence of oxidative stress within the MG. Elevated levels of HO-1 are also characteristic of cellular response to oxidative stress [46]. Recent studies have demonstrated the upregulation of HO-1 in heat-stressed cells [47], as it plays a crucial role in mitigating HS-induced apoptosis by activating antioxidant responses. In our study, we observed a significant rise in TAOC in the mammary vein during Mild- and Mod-HS, suggesting that the MG may enhance its antioxidant capacity by upregulating HO-1 levels as a protective mechanism against HS-induced oxidative damage.

## Conclusion

Our study investigated the impact of HS on MPF and mammary metabolism in dairy cows. During Mild-HS, we observed an increase in MPF, leading to a significant rise in mammary supply of oxygen and glucose. However, the available glucose was not fully utilized for milk synthesis, partially used for homeostatic regulation. In contrast, MPF decreased during Mod-HS, and the cows were unable to meet the metabolic demands of the substrates for synthesis of milk compositions. We also observed significantly decreased glucose uptake and efflux in the MG under Mod-HS. These findings indicate that HS influences mammary metabolism and synthesis largely by regulating nutrient supply through its impact on MPF. However, further investigations are warranted to clarify the mechanisms underlying these effects under different HS conditions.

## Data Availability

All data generated or analyzed during this study are included in this published article.

## Abbreviations

ALP: Alkaline phosphatase
ALT: Alanine aminotransferase
AST: Aspartate aminotransferase
ATP: Adenosine triphosphate
AV: Arterio-venous
BHB: β-Hydroxybutyrate
BUN: Blood urea nitrogen
eNOS: Endothelial nitric oxide synthase
GLP-1: Glucagon-like peptide-1
GLUT1: Glucose transporter 1
GSH-Px: Glutathione peroxidase
HDL: High-density lipoprotein
HIF-1α: Hypoxia-inducible factor 1α
HO-1: Heme oxygenase 1
HS: Heat stress
HSF: Heat shock transcription factor
HSP90: Heat shock protein 90
iNOS: Inducible nitric oxide synthase
IGF-1: Insulin-like growth factor-1
LDL: Low-density lipoprotein
MDA: Malondialdehyde
MG: Mammary gland
Mild-HS: Mild heat stress
Mod-HS: Moderate heat stress
MPF: Mammary plasma flow
MUN: Milk urea nitrogen
NO: Nitric oxide
No-HS: No heat stress
PFK: 6-Phosphofructokinase
RQUICKI: Revised quantitative insulin sensitivity check index
SEM: Standard error of the mean
SOD: Superoxide dismutase
TAOC: Total antioxidant capacity
VEGF: Vascular endothelial growth factor
